# Following the Crystallization of Amorphous Ice after Ultrafast Laser Heating

**DOI:** 10.1021/acs.jpcb.1c10906

**Published:** 2022-03-11

**Authors:** Marjorie Ladd-Parada, Katrin Amann-Winkel, Kyung Hwan Kim, Alexander Späh, Fivos Perakis, Harshad Pathak, Cheolhee Yang, Daniel Mariedahl, Tobias Eklund, Thomas J. Lane, Seonju You, Sangmin Jeong, Matthew Weston, Jae Hyuk Lee, Intae Eom, Minseok Kim, Jaeku Park, Sae Hwan Chun, Anders Nilsson

**Affiliations:** † Department of Physics, AlbaNova University Center, Stockholm University, Stockholm SE-10691, Sweden; ‡ Department of Chemistry, POSTECH, Pohang 37673, Republic of Korea; § SLAC National Accelerator Laboratory, 2575 Sand Hill Road, Menlo Park, California 94025, United States; ∥ Pohang Accelerator Laboratory, Pohang, Gyeongbuk 37673, Republic of Korea

## Abstract

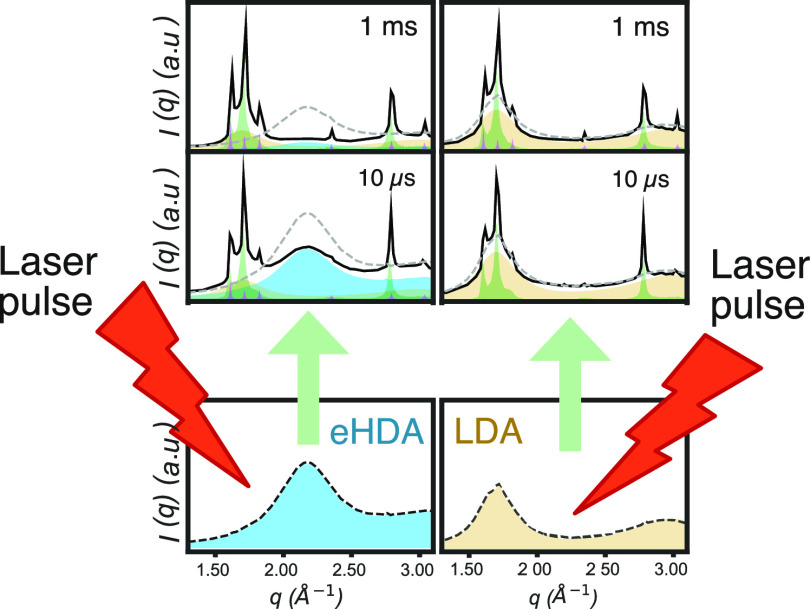

Using time-resolved wide-angle X-ray scattering, we investigated the early stages (10 μs–1 ms) of crystallization of supercooled water, obtained by the ultrafast heating of high- and low-density amorphous ice (HDA and LDA) up to a temperature *T* = 205 K ± 10 K. We have determined that the crystallizing phase is stacking disordered ice (*I*_sd_), with a maximum cubicity of χ = 0.6, in agreement with predictions from molecular dynamics simulations at similar temperatures. However, we note that a growing small portion of hexagonal ice (*I*_h_) was also observed, suggesting that within our timeframe, *I*_sd_ starts annealing into *I*_h_. The onset of crystallization, in both amorphous ice, occurs at a similar temperature, but the observed final crystalline fraction in the LDA sample is considerably lower than that in the HDA sample. We attribute this discrepancy to the thickness difference between the two samples.

## Introduction

Water crystallization is a phenomenon of utmost importance in a variety of areas, such as cloud formation,^1−3^ food preservation,^4,5^ and planetary sciences.^6^ Nevertheless, the early stages of water crystallization are not yet fully understood, especially in the temperature range between ca. 160 K^7^ and 232 K^8^ (often called “no man’s land”). The reason behind this is that water crystallizes spontaneously as either end of the regime is approached, thus making it challenging to induce and probe crystallization within no man’s land. However, different approaches have been used in an attempt to circumvent this problem and allow for experimental studies. By heating crystalline^9^ and amorphous ice,^7,10−15^ crystallization rates have been studied in the lower temperature region of “no man’s land” (*T* = 134–160 K).^6,13,14^ Decreasing the sample size has been another approach as it hinders crystallization, allowing for the supercooling of water. Studies then use either nanoconfinement of water^16−23^ or cooling of micrometer-^24−28^ or nanometer-sized^29,30^ droplets. Particularly, the use of microdroplets has allowed for the exploration of the nucleation rates down to 227 K,^24^ whilst nanodroplets have helped explore temperatures down to 200 K. However, nanodroplets are affected by their high internal pressure and large surface to volume ratio, making it hard to connect their behavior to that of microdroplets.^31^

In a more recent study, transient heating of thin water films allowed for the estimation of nucleation rates at temperatures of 188 K < *T* < 230 K.^9^ In this study, a maximum nucleation rate was observed at a temperature of 216 K ± 4 K, in agreement with nanodroplet experiments.^30^ Importantly, it cannot be excluded that the observed crystallization might be surface-induced or even heterogeneous, as evidenced by recent studies of crystallization in nanodroplets between *T* = 233 K and *T* = 235 K.^32^

**Figure 1:**
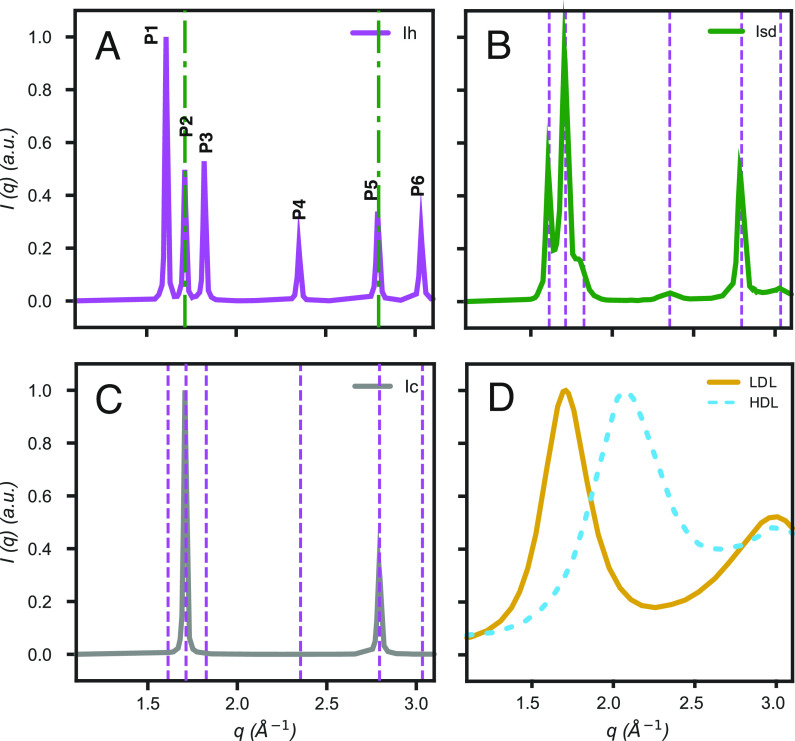
Typical diffraction patterns of (A) hexagonal ice (*I*_h_), (B) an example of stacking disordered ice (*I*_sd_) with 50% cubicity, (C) cubic ice (*I*_c_), and (D) HDL (dashed blue line)^15^ and LDL (yellow continuous line).^38^ Marked in dashed purple vertical lines are the peak positions of *I*_h_, and in dashed-dotted green lines the peak positions of *I*_c_. In Figure A, we have numbered the different peaks present in *I*_h_, from P1 to P6, of which, P2 and P5 are the peak positions shared between *I*_h_ and *I*_c_. Crystalline ice (A to C) scattering patterns were calculated using the FAULTS diffraction simulation software,^39^ following the same procedure as with DIFFaX.^35^

Whilst there has been a large focus on crystallization rates, it is also of interest to understand the nucleating crystal structure in “no man’s land”. The most stable crystalline phase of water at ambient pressure is that of hexagonal ice, *I*_h_, (Figure 1A). However, a different polytype has also been reported, that is, stacking disordered ice, *I*_sd_, (Figure 1B), which consists of randomly intercalated layers of cubic and hexagonal sequences. This crystalline form was originally referred to as cubic ice, *I*_c_,^33,34^ (Figure 1C). Nevertheless, it is evident from the X-ray scattering patterns, such as that shown in Figure 1B, that whilst it does display peaks related to a cubic phase (Figure 1C), it retains features of *I*_h_, especially the peak denoted as P1 (Figure 1A).^35^ One of the reasons why *I*_sd_ was often called *I*_c_ was because pure *I*_c_ had not been experimentally produced. Recently, two groups independently obtained pure *I*_c_, showing the typical diffraction pattern of a cubic structure (Figure 1C),^36,37^ without any of the additional features of *I*_sd_.

Experimentally, *I*_sd_ has been observed as the crystallizing form either at temperatures above 225 K or below 200 K.^2,35,40−42^ Whilst for temperatures between these two extremes it is only through molecular simulations^43−47^ that *I*_sd_ has been identified as the initial crystalline structure. Both through experiments and simulations,^35,40,42^ it has also been noted that the cubicity of *I*_sd_ decreases over time both at constant^42^ and increasing temperature,^35^ leading to annealing into *I*_h_, which is the thermodynamically stable phase.^42^

Recently, two different studies^15,48^ have reported on the crystallization of ice directly from a low-density noncrystalline phase (LDN). By measuring in situ X-ray diffraction during rapid decompression of high-pressure ice,^48^ a transition to an LDN between 140 and 165 K was observed. Eventually, the sample crystallized into *I*_sd_. On the other hand, X-ray scattering studies, combined with a laser-induced T-jump,^15^ indicated a discontinuous transition between high and low-density liquid water (HDL and LDL, respectively) at 205 K ± 10 K, prior to the onset of crystallization, that is, within a time frame between 8 ns and 1 μs. This transition was observed in the q-region between 1.1 and 2.5 Å^–1^, where the first-order peaks of both liquids are clearly observable: HDL (ca. 2.1 Å^–1^) and LDL (1.7 Å^–1^) (Figure 1B).

By looking at the nano- to microsecond scale, it was possible to open a window to observe the early stages of ice formation.^15^ Therefore, we have taken a deeper inspection into the later delays (10 μs onwards), from the same experiment, to explore the initial crystallizing forms in “no man’s land”. Within the observed timescale (10 μs–1 ms), water crystallizes in a stacking disordered manner with a high cubicity, in line with the results of Amaya et al.^40^ This cubicity decreases over time, similar to previous experiments.^21,23−25^ Whilst *I*_sd_ is the main crystal structure, we observe that a small proportion of *I*_h_ was also present, suggesting that a fraction of the crystallites started transforming into this form.^49,52^

## Methods and Materials

The experiments were conducted at the XSS-FXS beamline of PAL-XFEL, with 25 fs X-ray pulses with a mean energy of 9.7 keV and are described in detail by Kim et al.^15^ Here, we highlight some of the most important details.

### Sample Preparation

In brief, amorphous ice samples were prepared inside a 0.1 mm-thick Cu-grid using a material testing machine (Zwick, Z100 TN).^12,15,38,53^ The grid was filled with ultrapure deionized water and precooled over liquid nitrogen to form crystalline ice. After cold-loading the grids into a piston cylinder setup and covering the grids with a thin In-foil, unannealed high-density amorphous ice was formed by pressurizing to 1.6 GPa at 100 K. The samples were then heated and annealed at 160 K and 1.1 GPa, resulting in very-high-density amorphous ice. For transformation into equilibrated high-density amorphous ice (eHDA) or low-density amorphous ice (LDA), the samples were decompressed at 139.5 K to 0.08 and 0.01 GPa, respectively, and subsequently quenched to 80 K. The resulting eHDA and LDA samples were decompressed to ambient pressure, stored, and transported under liquid nitrogen temperatures to PAL-XFEL, South Korea.

### Laser and X-ray Parameters

We utilized an infrared-pump X-ray probe scheme to obtain time-dependent single-shot X-ray scattering patterns. Samples were loaded on a sample environment consisting of a liquid N_2_-flow cryostat capable of operating in vacuum between 80 K and room temperature.

The samples were then pumped by a 100 fs infrared (IR) pulse with a 2 μm wavelength, which excited a combination of O–H stretch and H–O–H bending modes that increased the temperature of the sample in ca. 20 ps,^54,55^ resulting in an ultrafast temperature-jump (T-jump) of about 90 K, in the case of HDA, and of 60 K for LDA,^15^ resulting in a final temperature of around 205 ± 10 K. Scattering images were taken at different delays to capture the whole T-jump. The images were taken in the wide-angle regime covering a *q*-range of 0.1–3.2 Å^–1^, allowing for the observation of the first diffraction peaks of the high- and low-density structures. Laser-off images were acquired and used as a reference, and for obtaining the time-resolved difference X-ray scattering patterns.

### Data Analysis

The proportions of the different components (HDL, LDL, and crystalline ice) were estimated by fitting the time-dependent difference scattering curves, obtained by taking the difference between the *I*(*q*) curves measured before and after laser excitation (see the Supporting Information Figure S1).

We particularly focused on the relative amount of cubic vs hexagonal stacking. Different parameters were considered for this objective: the fraction of *I*_h_ (obtained from the fittings), the degree of cubicity, the ratio between P2 and P1, and the intensity of P4 at ca. 2.34 Å^–1^.

The degree of cubicity (χ) is defined as follows:^35,41,42,56^
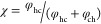
1where φ_hc_ is the probability of a cubic layer to form over a hexagonal layer, and φ_ch_ is the probability of a hexagonal layer to form over a cubic layer. To obtain the P2:P1 ratio and the intensity of P4, the liquid (HDL and LDL) fractions were subtracted from the postpump shots (Figure S3). The P2:P1 ratio quantifies the degree of cubicity as P1 is unique to *I*_h_, whereas P2 is shared between *I*_h_ and *I*_c_. In pure *I*_h_, the P2:P1 ratio is approximately 0.50,^50^ and higher values indicate a larger amount of cubic stacking.^51,52^ In the case of P4, this peak is only present in pure *I*_h_; therefore, it can be used as a marker for hexagonal stacking.^50^

Finally, from the obtained crystalline fraction, the nucleation and growth rates were roughly estimated for both samples, as detailed in the Supporting Information of Kim et al.^15^ using the following equation:
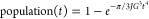
2where *J* is the nucleation rate, *G* is the growth rate, and *t* is time. It should be noted that the values of *J* and *G* vary with temperature.

We fitted the crystallization curves only up to 100 μs, as after this delay, cooling dominates.^15^ In the case of the eHDA sample, this leads to an apparent decrease in the crystalline fraction because of the increase in the LDL fraction. The fits were performed by leaving the growth rate as a free parameter, but fixing *J* to the value estimated by Kimmel et al.^9^ for 200 and 205 K, for the LDA and eHDA samples, respectively, as these are the estimated reached temperatures.^15^

## Results and Discussion

**Figure 2:**
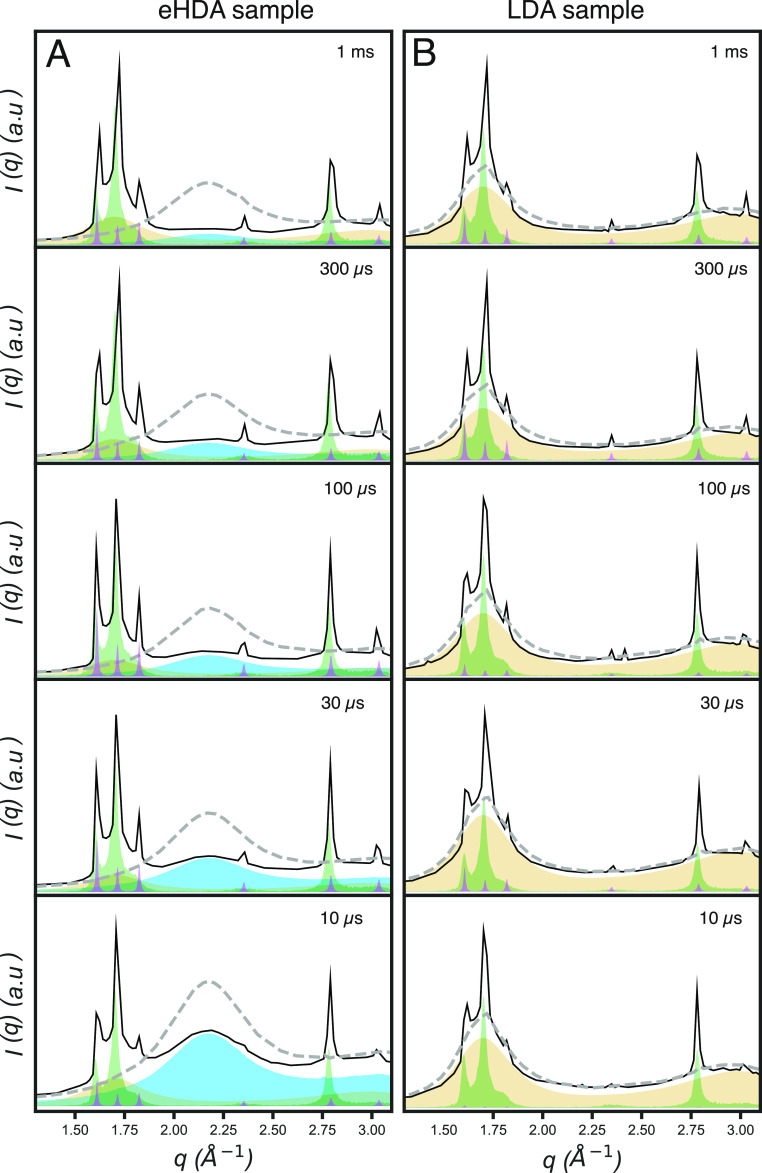
Time-resolved wide-angle X-ray scattering patterns showing the progression of crystallization from 10 μs to 1 ms. (A) eHDA at a base temperature of 115 K and (B) LDA at a base temperature of 140 K. Prepump scattering is shown as a gray dashed line and the postpump as a black continuous line. Contributions of the HDL are indicated in blue, LDL in yellow, *I*_sd_ in green, and *I*_h_ in purple.

We performed measurements after different delay times between 8 ns and 1 ms. However, crystallization is only evident from 3 μs onward, and it is only from 10 μs that a good enough fit was possible. Therefore, we focus on these later delays where ice Bragg peaks are observable in both eHDA and LDA samples (Figure 2).

From the scattering patterns, it is already evident that in the eHDA sample (Figure 2A), the HDL fraction decreases over time, as the broad peak centered at ca. 2.1 Å^–1^ almost disappears by the 1 ms delay time. Crystallization, as expected, has the opposite behavior with increasing intensity of the crystalline Bragg peaks. In the LDA sample (Figure 2B), however, the decrease in the LDL fraction is not as dramatic as that of the HDL in the eHDA sample. However, crystallization increases as particularly evidenced by the intensity of P4 and P6 (ca. 2.3 and 3 Å^–1^).

We further fitted the difference scattering patterns with a linear combination of HDL, LDL, and crystalline ice (Figure S1). Importantly, for the crystalline ice fraction, we initially considered stacking disordered ice (*I*_sd_) only, but it frequently underestimated the sharpness and height of P3 and P4, as shown in Figure S2A. Therefore, we decided to use a combination of simulated pure hexagonal ice (*I*_h_) and *I*_sd_, resulting in a better fit (Figure S2B). The improvement of the fit by the introduction of the diffraction pattern of *I*_h_ suggests that part of the *I*_sd_ has started to anneal into *I*_h_ and that we thus have a combination of two different crystalline ice.

**Figure 3:**
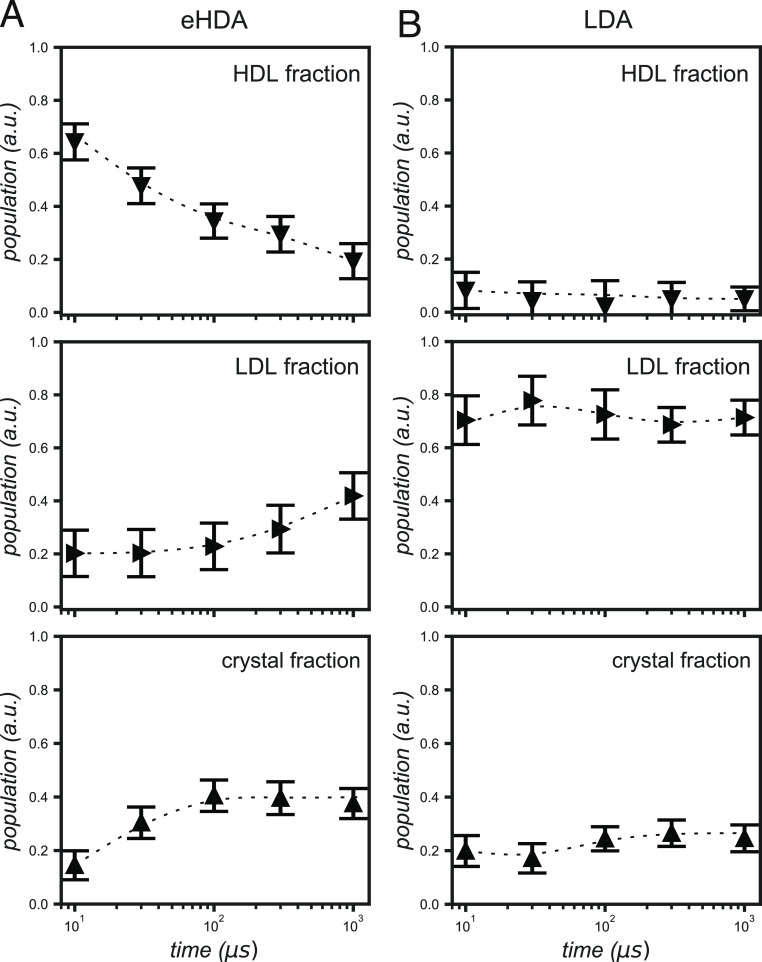
Time-dependent population changes from 10 μs to 1 ms. (A) Fractions of HDL, LDL, and crystalline ice taken from the fits of the (A) eHDA samples and of the (B) LDA samples. Downward triangles represent the HDL fractions, the right triangles the LDL fractions, and the upward triangles the crystalline (*I*_h_ + *I*_sd_) fraction. Bars indicate the error of the fit. Dotted lines are added just as a guide to the eye.

In the eHDA sample (Figure 3A), we observe that the HDL fraction decreases continuously as it converts to LDL. However, we do not see a parallel increase in the LDL fraction. In fact, we see that the LDL fraction initially remains stable at a population fraction of ca. 0.20 and only increases from 100 μs onward. Nevertheless, the crystal fraction initially increases and then stabilizes at 100 μs. Consequently, we can infer that the apparent discrepancy between the LDL and HDL behavior is related to the progressing crystallization. Between 10 and 100 μs, the LDL constantly crystallizes; thus, the observable amount of LDL instantly decreases, even though we know that the HDL continuously transforms into LDL. From Kim et al.^15^ we know that considering the thermal diffusivity of our samples, we can estimate that prior to 100 μs, the temperature of the sample is stable. However, after 100 μs, the sample starts cooling again^15^ because of the rest of the sample being at 115 K, resulting in a higher viscosity that hampers crystallization, thus allowing us to see the growth in the LDL fraction once more.

In the LDA sample (Figure 3B), the crystalline fraction initially increases between 30 and 100 μs but remains stable after this delay, which is also attributable to the estimated cooling. Whilst the overall trend is similar to that of the eHDA sample, we note that the final crystal fraction is considerably smaller and that consequently the liquid fraction is higher. The reduced crystalline fraction is most likely caused by our LDA samples being thicker (>55 μm) than those of eHDA (35–55 μm). This larger thickness causes the front part of the sample to absorb more heating light than the back, thus creating a larger temperature difference between the two sides of the sample, which can lead to a faster cooling from 100 μs onward. Moreover, we cannot exclude that the transition from HDL to LDL has an exothermic character, causing a further temperature increase, thus accelerating crystallization in the eHDA sample. Finally, we also observe a minor fraction of HDL ((Figure 3B) but with error bars going down to negative values at most delay times. Thus, we do not consider it to be of particular significance.

We also note that, even at 1 ms, both samples retain fractions of both LDL and HDL, in agreement with previous studies where a coexistence between crystalline ice and supercooled water has been reported even after several seconds.^6,57−60^ These results differ from those of Kim et al.^15^ in that study, and it was assumed that the sample was completely crystalline after 1 ms. However, the focus of that study was on the earlier delays (before 10 μs), where the HDL to LDL transition occurs, and the proportion of crystalline ice is either nonexistent or insignificant. Thus, the assumption of a fully crystalline sample at 1 ms is consistent with the conclusions there reached.

**Figure 4:**
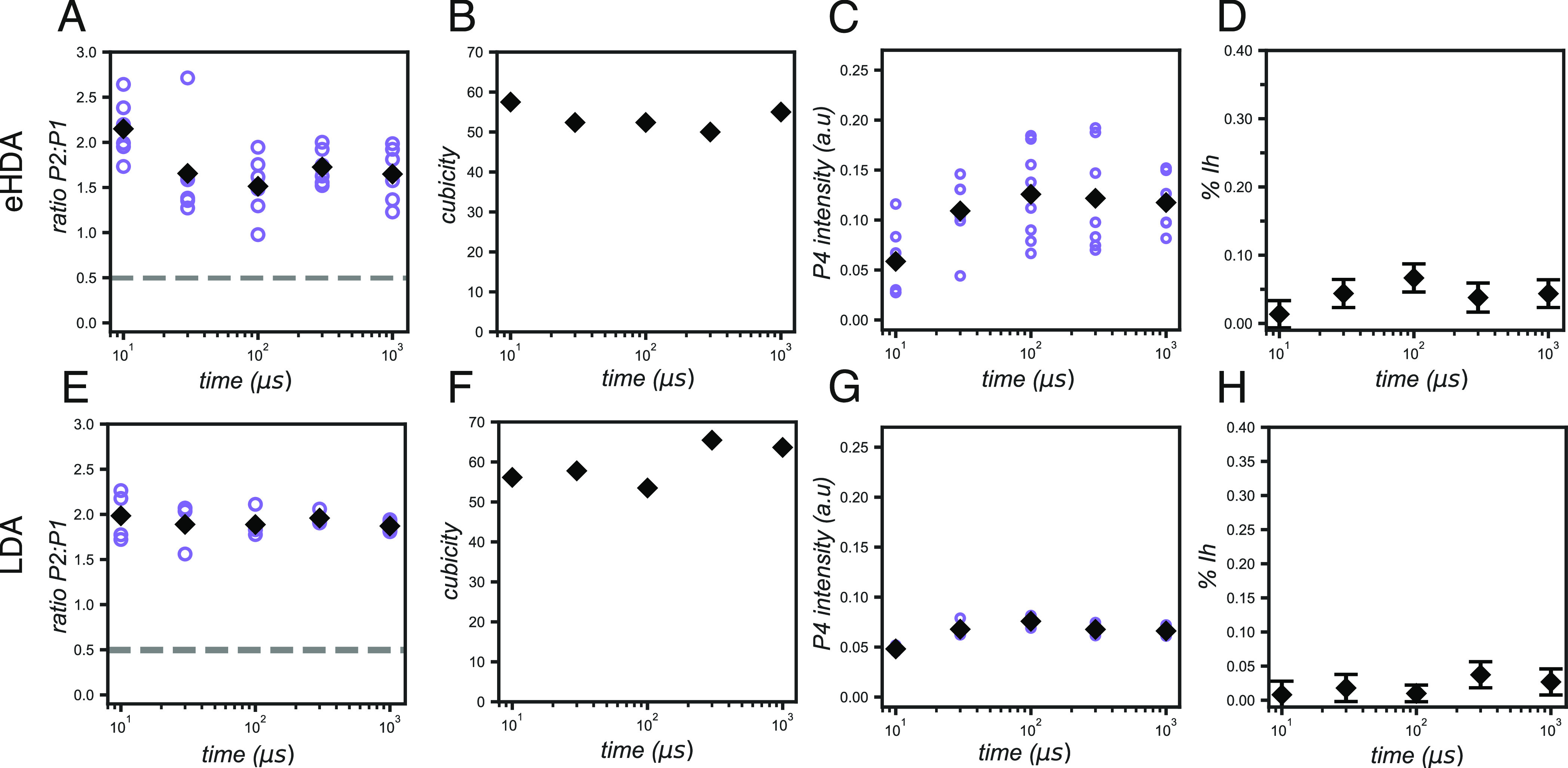
Ratio of P2:P1, cubicity (χ), intensity of P4, and % of *I*_h_ as a function of the delay time of (A to D) eHDA samples and (E to H) LDA samples. A dashed line has been added to the plots of the P2:P1 ratios (A and E) to indicate the ratio observed in a pure *I*_h_ scattering pattern. Values higher than this ratio (ca. 0.5) indicate a higher proportion of cubicity. Purple circles in the plots A, C, E, and G are the values for individual samples, and the black diamonds are the average of all the measurements. Bars in plots D and H indicate the error of the fit.

As mentioned before, the main crystal form obtained was that of *I*_sd_, and from the fitted scattering patterns, we were able to estimate the relative amount of cubic and hexagonal stacking present in the sample. We used different approaches for this purpose. We evaluated the ratio between P2 and P1, the degree of cubicity, the intensity of P4, and the fraction of *I*_h_ (Figure 4).

In the eHDA sample, the P2:P1 ratio (Figure 4A) initially decreases and stabilizes after 100 μs. As expected, the intensity of P4 consequently increases and stabilizes after the same time delay (Figure 4B), suggesting that the relative amount of hexagonal stacking increases at the expense of the cubic stacking. Similar trends are observed in the cubicity and fraction of *I*_h_ (Figure 4C, D).

In the LDA sample, the P2:P1 ratio remains at a similar level to that of eHDA at 10 μs (Figure 4E), whilst the intensity of P4 increases slightly between 10 and 100 μs, after which it remains stable (Figure 4F), suggesting an initial minor increase in hexagonal packing. Notably, the degree of cubicity increases slightly at higher delay times, alongside the fraction of *I*_h_ (Figure 4F, H), which is surprising as we would expect them to be inversely related, not to mention that this trend was not observed in the P2:P1 ratio, nor in the P4 intensity. The behavior of the cubicity and *I*_h_ might then be an effect of the fitting itself. In the LDA scattering patterns, P5 has a strong intensity (Figure S3), sometimes almost equal to P2, suggesting a preferred orientation of the crystal, which is not considered in our *I*_sd_ simulations. Therefore, our fitting could be overestimating the amount of *I*_h_ in an attempt to reach the higher intensity of P5 in the experimental data. As a consequence, other *I*_h_ peaks would have higher intensities than those experimentally obtained, and a higher cubicity *I*_sd_ would then be necessary to compensate. Nevertheless, given that the P2:P1 ratio and P4 intensities are in agreement, we consider that our fits are still a good indicator of the overall trend of increase in hexagonal stacking up to 100 μs.

Lastly, the cubicities here estimated are in agreement with previous molecular simulations at similar temperatures,^43,45,61^ reporting values between 0.60 and 0.70. Experimentally, cubicities have mostly been reported to reach a maximum of 0.5,^42,62^ except for the work by Malkin et al.,^35^ who reported a cubicity of 0.78 when recrystallizing ice II into ice I. Amaya et al.^40^ also estimated a cubicity close to 0.80 at 225 K, which they attribute to both the low temperature and the faster crystallization observed in nanodroplets.^30^

Finally, we performed fits based on the nucleation data from Kimmel et al.^9^ and the growth rate data from Xu et al.^63^ to evaluate them in the current system. By fixing the nucleation rate (*J*) to the values from Kimmel et al.^9^ at the estimated temperatures, for each sample (205 and 200 K for eHDA and LDA, respectively), we obtained, for eHDA, a growth rate close to that estimated by Xu et al. at 225 K^63^ (see Table S1), that is, 20 K above the estimated T-jump. In comparison, the fit for the LDA sample resulted in a growth rate close to that at 235 K, that is, 35 K above the estimated T-jump. The temperature difference between nucleation rate *J* and growth rate *G* can be assumed to originate from the heat release during the nucleation process, leading to crystal growth occurring at a different temperature. This is further reinforced by the estimated temperature increase because of crystallization between 17.5 and 39.5 K (see the Supporting Information), assuming that there is no heat release to the environment because of the timescales studied here.^64,65^ The estimated temperature increase is in the same order of magnitude as the increase in temperature assumed by using the growth rates at 20 and 36 K higher than the nucleation rates.

**Figure 5:**
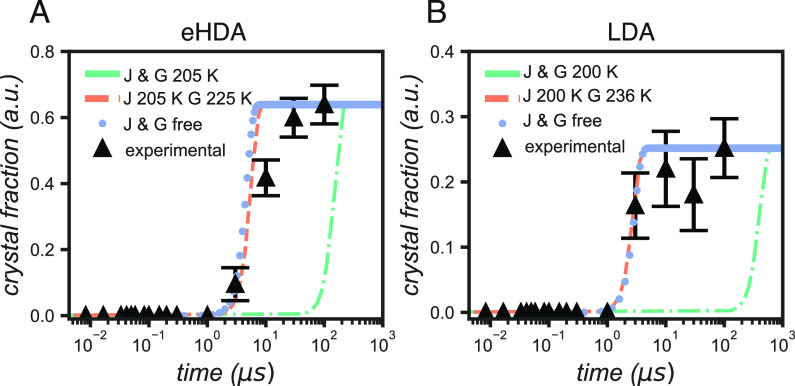
Crystallization curves of (A) the eHDA sample and (B) the LDA sample. Black triangles are the experimental data. Dotted blue line is the corresponding fit where the nucleation rate, *J*, was fixed to the values from Kimmel et al.^9^ at 205 and 200 K for the eHDA and LDA samples, respectively. Dashed orange line shows the theoretical crystallization curve using the same *J*(9) as before, and the growth rate, *G*, from Xu et al.^63^ closest to the estimated *G* from the blue fit (i.e., the *G* at 225 and 235 K for the eHDA and LDA samples, respectively). Dashed-dotted green line is the theoretical crystallization curve using *J*(9) and *G*(63) at the estimated temperature for each sample.

After the first crystallization point at 3 μs, the experimental data follow a slower crystallization rate than the fits (Figure 5). In the case of the eHDA sample, this can be explained by crystallization not happening in the bulk, but only in the new LDL domains. As the crystalline material grows within these domains, crystallites can eventually impinge on each other, slowing the growth rate. In the case of the LDA sample, we could expect bulklike crystallization as the whole sample is considered to have converted into LDL. Nevertheless, we have estimated that LDA samples have a larger thickness (>55 μm) than the eHDA (35–55 μm), leading to further cooling from the back side of the sample, which has not been heated to the same degree. This cooling then reduces the growth rate as the viscosity of the liquid increases, hindering crystallization. Further evidence of the hindering of crystallization can be found in the final crystalline fraction. The LDA samples only reach a fraction of ca. 0.25, in comparison to the eHDA samples that reached almost 0.6.

## Conclusions

Within the timescales evaluated in this study, we observe that the crystallization of LDL coming from heating either HDA or LDA occurs in the microsecond scale in a stacking disorder form (*I*_sd_) with a large cubicity (0.5–0.65), which decreases over time. Nevertheless, even in these short timescales, some of the ice anneals into *I*_h_, the thermodynamically preferred crystalline state,^42^ leading to a mix of *I*_sd_ and *I*_h_. The crystallization of the LDA sample stagnated from 30 μs onward, thus reaching a lower final crystalline fraction than the eHDA sample. We ascribe this to the larger thickness of the LDA samples. Finally, while the first crystallization points are correctly fitted by using literature nucleation values at the estimated sample temperatures, the estimated growth rate is equivalent to values at higher temperatures, which could be attributed to the heat release during nucleation.

## Supporting Information Available

Difference scattering curves and their fits, calculation of the P4 intensities, P2:P1 ratios, nucleation and growth rates used in Figure 5, and estimation of the temperature increase due to crystallization (PDF)
