# Supplementary material for: Following the Crystallization of Amorphous Ice after Ultrafast Laser Heating
Source: J Phys Chem B. 2022 Mar 11;126(11):2299–307. doi: 10.1021/acs.jpcb.1c10906 (PMC8958512; doi:10.1021/acs.jpcb.1c10906)
Supplement: Supplementary file 1 — jp1c10906_si_001.pdf [file jp1c10906_si_001.pdf]

Following the Crystallisation of Amorphous Ices after Ultrafast Laser Heating

Marjorie Ladd-Parada[1], Katrin Amann-Winkel[1], Kyung Hwan Kim[2], Alexander Späh[1,3], Fivos Perakis[1], Harshad Pathak[1], Cheolhee Yang[2], Daniel Mariedahl[1], Tobias Eklund[1], Thomas. J. Lane[3], Seonju You[2], Sangmin Jeong[2], Matthew Weston[1], Jae Hyuk Lee[4], Intae Eom[4], Minseok Kim[4], Jaeku Park[4], Sae Hwan Chun[4], and Anders Nilsson[1]*

[1]Department of Physics, AlbaNova University Center, Stockholm University, SE-10691 Stockholm, Sweden

[2]Department of Chemistry, POSTECH, Pohang 37673, Republic of Korea

[3]SLAC National Accelerator Laboratory, 2575 Sand Hill Road, Menlo Park, California 94025, USA

[4]Pohang Accelerator Laboratory, Pohang, Gyeongbuk 37673, Republic of Korea

Supplementary materials

Difference Scattering Curves, Fitting Analysis, and Population Changes

Time-dependent difference scattering curves were obtained by taking the difference between the I(q) curves measured before and after laser excitation These curves were then fitted by a combination of depletion of HDL, formation of LDL, and formation of crystalline ice, similarly to Kim, KH, Amann-Winkel, K, et al.[15]

$$\Delta I_{theory}(q) = -C_{HDL} I_{HDL}(q) + C_{LDL} I_{LDL}(q) + C_{crystal} I_{crystal}(q) \qquad (S1)$$

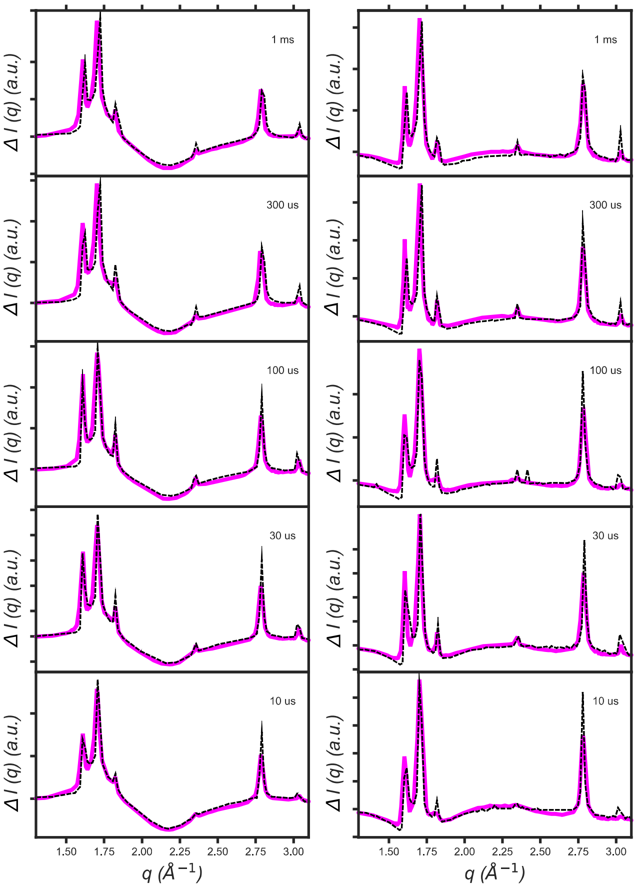

Fig. S1 Example of the difference between post and pre-pump (dashed line) x-ray pattern and the fitting using equation 1 of the Supplementary Materials (continuous pink line). The data shown here belongs to the eHDA sample at 115 K after a 1 ms delay.

For HDL, we used the scattering pattern measured before the pump at the same spot, for LDL, we used the reference scattering pattern that was obtained from a previous study[67], and for crystalline ice we initially considered a simulated stacking disordered ice (I_{sd}) but it frequently underestimated the sharpness and height of peaks 3 and 4 (P3 and P4), as shown in Fig S2 A. Therefore, we decided to use a combination of simulated pure hexagonal ice (I_h) and I_{sd}, i.e. $C_{crystal}I_{crystal} = C_{I_{sd}}I_{I_{sd}} + C_{I_h}I_{I_h}$, resulting in a better fit (Fig S2 B).

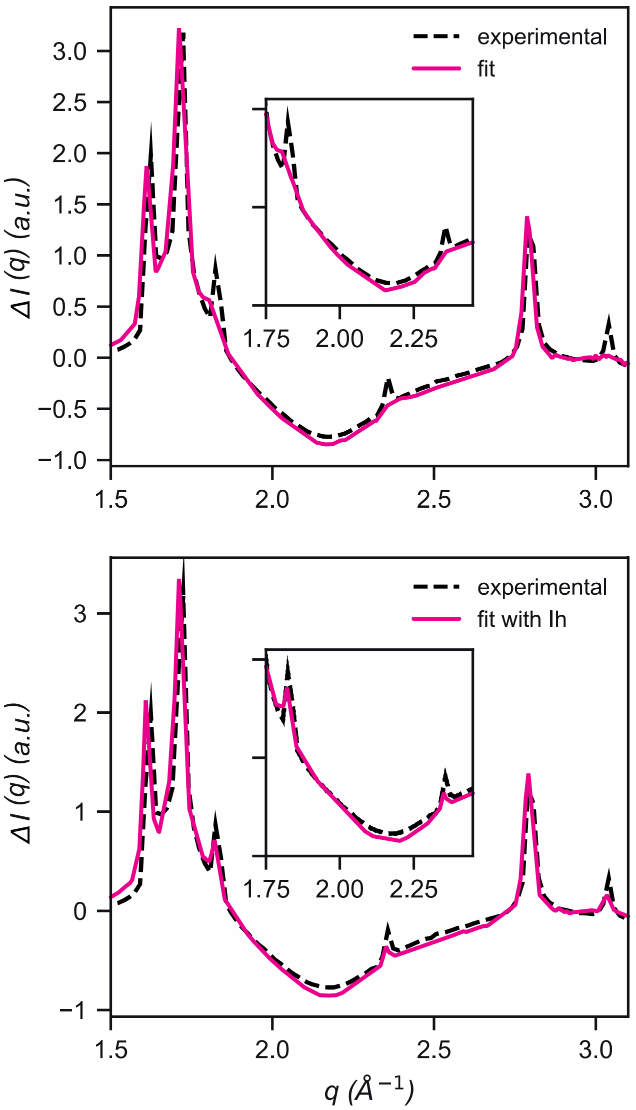

Fig. S2 Example of the experimental difference x-ray diffraction pattern (dashed black line), fitted with only A) Isd and with a combination of B) Isd and Ih (magenta continuous line). In both images there is an inset with the same data, zooming in the region between 1.75 to 2.4 $Å^{-1}$ where the fit of P3 and P4 can be observed in better detalil. The plots here correspond to the eHDA sample after 1 ms delay time post-pump.

We note that for the LDA sample, the equation had to be adjusted accordingly, as the low-density fraction is the one decreasing overtime:

$$\Delta I_{theory}(q) = C_{HDL}I_{HDL}(q) - C_{LDL}I_{LDL}(q) + C_{crystal}I_{crystal}(q) \qquad (S2)$$

For the fitting of the LDA sample, we used the same reference scattering patterns as before, except for the HDL reference where we used the average of the pre-pump values of the 10 ms delay time.

The I_{sd} and I_h simulated scattering patterns were calculated using the FAULTS diffraction simulation software[39] following the same procedure based on the probabilities of a cubic or a

hexagonal stacking, as previously done with DIFFaX[35]. A maximum likelihood estimation using a least square methods was employed, using the MINUIT software package[68].

Calculation of P4 intensities and P2:P1 ratios

To compare the P4 intensities and calculate de P2:P1 ratios, the LDL and HDL fractions were subtracted from the x-ray diffraction to create a flat baseline. These fractions, or contributions, were obtained from the fitting described above. Once the subtraction was done, the plots were normalised to the intensity of P2.

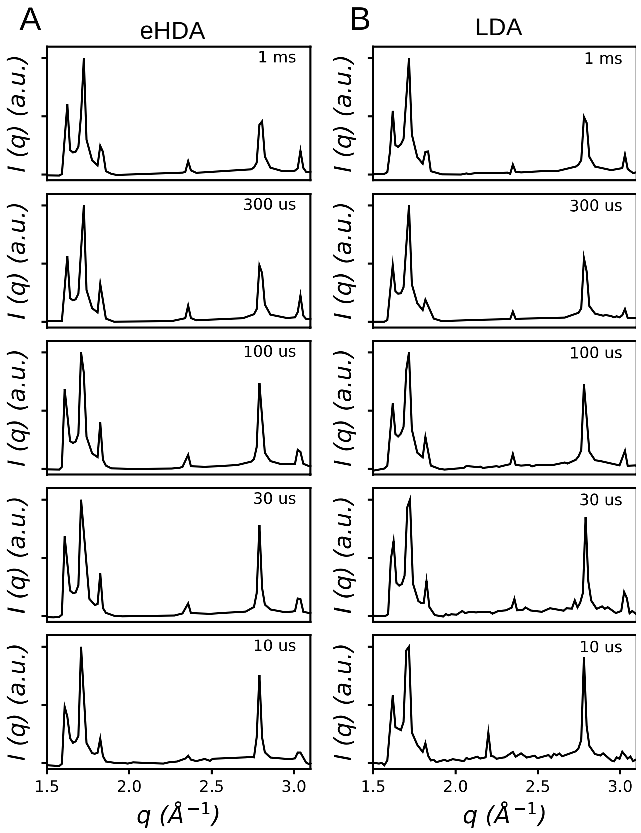

Fig. S3 Normalised wide angle x-ray scattering patterns of heated A) eHDA and B) LDA samples, from which the liquid (HDL and LDL) contributions have been subtracted. Here we show the delay times between 10 ms and 1 ms.

Nucleation and growth rates

Table S1 Shows the nucleation (J) and growth (G) rates estimated for the crystallisation curves of the eHDA and the LDA samples.

The free fitting parameters are obtained by leaving both J and N free, whilst for the other fits, we fixed J to the value of either 205 K (eHDA sample) or 200 K (LDA sample) as these are the estimated T jumps. For comparison, we are reporting the estimated growth rate for both temperatures for both amorphous ices.

Table S1 Nucleation and growth rates of eHDA and LDA

Parameter	eHDA	LDA
	Fixing J to 205 K	
**J ($cm^{-3}s^{-1}$)**	$3.0*10^{20}$ *	$3.0*10^{20}$ *
**G($cm\ s^{-1}$)**	$1.0*10^{-8} \pm 0.2*10^{-8}$	$1.9*10^{-8} \pm 0.3*10^{-8}$
	Fixing J to 200 K	
**J ($cm^{-3}s^{-1}$)**	$6.0*10^{19}$ *	$6.0*10^{19}$ *
**G($cm\ s^{-1}$)**	$1.5*10^{-8} \pm 0.2*10^{-8}$	$2.8*10^{-8} \pm 0.3*10^{-8}$

* Taken from Kimmel, et al.[9]

Estimation of the temperature increase due to crystallisation

We estimated the temperature increase by using the heat balance equation of our sample:

$$xm\Delta H_m = xm\Delta T C_{ice} + (1-x)m\Delta T C_{liq} \qquad (S3)$$

where x is the fraction of crystalline material, m is the mass of the sample, DH_m is the latent heat of melting – 333 kJ/kg – and C_{ice} and C_{liq} are the heat capacities of ice and water corresponding to 2.10 kJ/kgK and 4.22 kJ/kgK, respectively.

References

(67) Perakis, F.; Amann-Winkel, K.; Lehmkühler, F.; Sprung, M.; Mariedahl, D.; Sellberg, J. A.; Pathak, H.; Späh, A.; Cavalca, F.; Schlesinger, D.; et al. Diffusive Dynamics during the High-To-Low Density Transition in Amorphous Ice. *Proc. Natl. Acad. Sci. U. S. A.* **2017**, *114* (31), 8193–8198. https://doi.org/10.1073/pnas.1705303114.

(68) Dembinski, H.; Ongmongkolkul, P.; Deil, C.; Hurtado, D. M.; Schreiner, H.; Feickert, M.; Andrew; Burr, C.; Rost, F.; Pearce, A.; et al. Scikit-Hep/Iminuit: V2.0.0. **2020**. https://doi.org/10.5281/ZENODO.4310361.
